# Open-source electrochemical cell for *in situ* X-ray absorption spectroscopy in transmission and fluorescence modes

**DOI:** 10.1107/S1600577524000122

**Published:** 2024-02-02

**Authors:** Hiram Lopez-Astacio, Brenda Lee Vargas-Perez, Angelica Del Valle-Perez, Christopher J. Pollock, Lisandro Cunci

**Affiliations:** a Department of Chemistry and Physics, Universidad Ana G. Mendez at Gurabo, Gurabo, Puerto Rico, USA; b Department of Chemistry, University of Puerto Rico at Rio Piedras, San Juan, Puerto Rico, USA; c Cornell High Energy Synchrotron Source, Wilson Laboratory, Cornell University, Ithaca, NY 14853, USA; Advanced Photon Source, USA

**Keywords:** electrochemical cells for *in situ* XAS, EXAFS, ORR catalysts

## Abstract

X-ray spectroscopy is a valuable technique for the study of many materials systems. Characterizing reactions *in situ* and *operando* can reveal complex reaction kinetics, which is crucial to understanding active site composition and reaction mechanisms. In this project, the design, fabrication and testing of an open-source and easy-to-fabricate electrochemical cell for *in situ* electrochemistry compatible with X-ray absorption spectroscopy in both transmission and fluorescence modes are accomplished via windows with large opening angles on both the upstream and downstream sides of the cell. Using a hobbyist computer numerical control machine and free 3D CAD software, anyone can make a reliable electrochemical cell using this design. Onion-like carbon nanoparticles, with a 1:3 iron-to-cobalt ratio, were drop-coated onto carbon paper for testing *in situ* X-ray absorption spectroscopy. Cyclic voltammetry of the carbon paper showed the expected behavior, with no increased ohmic drop, even in sandwiched cells. Chronoamperometry was used to apply 0.4 V versus reversible hydrogen electrode, with and without 15 min of oxygen purging to ensure that the electrochemical cell does not provide any artefacts due to gas purging. The XANES and EXAFS spectra showed no differences with and without oxygen, as expected at 0.4 V, without any artefacts due to gas purging. The development of this open-source electrochemical cell design allows for improved collection of *in situ* X-ray absorption spectroscopy data and enables researchers to perform both transmission and fluorescence simultaneously. It additionally addresses key practical considerations including gas purging, reduced ionic resistance and leak prevention.

## Abstract 2

An economical and easy-to-fabricate electrochemical cell for *in situ* X-ray absorption spectroscopy was developed, fabricated, and used to obtain XANES and EXAFS data for a catalyst for the oxygen reduction reaction. The experiments were run with and without oxygen purging using an attachment that avoids interactions between bubbles and the working electrode, and X-ray absorption spectroscopy data were obtained under applied potential and with and without gas purging, showing the capabilities of this electrochemical cell for *in situ* experiments.

## 1. Introduction

*In situ* characterization approaches have the potential to provide extensive insights into complex reaction dynamics (Yang, Feijóo *et al.*, 2023 ▸; Fabbri *et al.*, 2017 ▸; Timoshenko & Roldan Cuenya, 2021 ▸; Kerr *et al.*, 2022 ▸; Khare *et al.*, 2020 ▸; Cheaib *et al.*, 2019 ▸), which is an important step in learning about the composition of active sites and the processes that are involved in reactions (Yang, Louisia *et al.*, 2023 ▸). *In situ* conditions include the evaluation of catalytic activity while simultaneously characterizing a working catalyst during conditions that are representative of actual reactions (Li *et al.*, 2019 ▸). In this article, we are using the definition of ‘*in situ*’ as explained by Zhu *et al.* (2020 ▸). Electrochemical X-ray absorption spectroscopy (XAS) is an example of one of these important methods that allow one to obtain structural information, such as oxidation state and site symmetry of the absorbing atom as well as distances and identities of the absorber’s nearest neighbors (Penner-Hahn, 1999 ▸), at real working potentials (Gao & Abruña, 2014 ▸; Zhang *et al.*, 2019 ▸; Seok *et al.*, 2020 ▸; Yang *et al.*, 2019 ▸; Yang, Feijóo *et al.*, 2023 ▸). The high penetration of hard X-rays makes it possible to conduct an *in situ* analysis of catalysts within an electrochemical cell while applying different potentials (Yang *et al.*, 2019 ▸).

Recent work has demonstrated the critical need for characterizing electrochemical and electrocatalytic processes under *in situ* conditions (Feijóo *et al.*, 2023 ▸; Yang, Louisia *et al.*, 2023 ▸; Yang *et al.*, 2019 ▸), since only under such conditions can one be sure that the species identified are in fact relevant to the reaction being studied and not precursor, inactive or off-pathway materials. The flexibility of XAS matches well to the large variability of electrochemically active materials, which can vary greatly in terms of composition, size, required electrolyte, *etc*. A challenge, however, is housing these reactions in a sample cell that allows X-rays to probe the material of interest and subsequently reach a detector. A wide variety of sample cells exist and have been previously reported; these vary from relatively simple reservoirs with space for electrodes/electrolyte to much more advanced systems with features such as electrolyte circulators, gas-delivery capabilities and vacuum compatibility (Timoshenko & Roldan Cuenya, 2021 ▸). Every existing sample environment possesses its own set of strengths and weaknesses and, while it is unfortunately not possible for us to review all existing *in situ* electrochemical sample environments in this article, we will comment briefly on general gaps in the capabilities of the cells that currently exist. Due to geometric factors, many such cells are suitable only for fluorescence XAS measurements (Best *et al.*, 2016 ▸; Favaro *et al.*, 2017 ▸; Khare *et al.*, 2020 ▸); these types of sample cells are not suitable for electrodes where the atom of interest exists in high concentrations (*e.g.* solid metals) as fluorescence-detected spectra on those samples would yield self-absorbed/over-absorbed XAS data (Kerr *et al.*, 2022 ▸). Other sample environments are more flexible and can perform either fluorescence or transmission measurements (Cheaib *et al.*, 2019 ▸; Yang *et al.*, 2019 ▸; Russell & Rose, 2004 ▸; Binninger *et al.*, 2016 ▸), though we are aware of none that allow both collection modalities simultaneously. Moreover, all of the previously reported cells require fabrication protocols that necessitate access to machining facilities, thus rendering them inaccessible to research groups that lack access to such equipment/knowledge. The possibility of designing an open-source versatile electrochemical XAS cell that can be modified and fabricated using free software and economically and widely available hobbyist computer numerical control (CNC) machines persuaded the authors to fabricate, test and showcase this work. Significantly, beam time is provided at no cost for user groups. However, as remote beamline operation and data-collection methods advance, obstacles in sample preparation can swiftly emerge as the primary hindrance to researchers seeking access to synchrotron facilities. Our cell is fabricated using a CNC machine that comes at a remarkably affordable price of less than USD 200. The materials used are highly economical, and the design is open source, enabling anyone to manufacture it without requiring extensive design knowledge or substantial research grants. This inclusivity is especially relevant in regions around the world where such conditions are prevalent.

Herein, a new open-source electrochemical cell design was developed to improve the cell’s reliability and performance for *in situ* electrochemical XAS experiments in transmission and fluorescence modes. By arranging the positions of the working electrode, counter electrode and reference electrode, we accomplished an electrochemical XAS cell with low ionic resistance and an attachment to purge the solution with gas while avoiding the interaction between bubbles and the electrodes and X-ray beam. In addition, by creating a 45° angle slope on both sides of the X-ray receiving window, a large (12.7 mm × 12.7 mm) area of access to the sample was provided, enabling the use of both transmission and fluorescence XAS modes. The ability of this cell to perform both transmission and fluorescence measurements at the same time presents numerous advantages. Foremost, it allows for a single sample environment and geometry to be used regardless of the electrode material and element of interest, and eliminated the need to change sample cells to accommodate different concentrations of the absorbing atoms. Moreover, this sample cell also enables new combined experiments such as simultaneous transmission/high-energy resolution fluorescence detected (HERFD) XAS (Lafuerza *et al.*, 2020 ▸), which would allow both precise metrical information (via transmission EXAFS) and high-resolution electronic structure information (via the HERFD near edge) to be obtained during the same energy scan of an experiment. Finally, the low cost and ease of fabrication permit this sample cell to be produced and used by researchers who lack access to large-scale machining facilities, and it thus may be of particular interest to groups at primarily undergraduate institutions, minority-serving institutions and groups located in developing countries.

Electrochemical cells made of acrylic, polyether ether ketone (PEEK) and polytetra­fluoro­ethyl­ene (PTFE) were fabricated with an economical hobbyist CNC machine, while *in situ* XAS and electrochemical experiments were performed to characterize the electrochemical XAS cell. The blueprints and open-source files are provided in this publication.

## 2. Experimental section

### 2.1. *In situ* XAS experiments

*In situ* XAS data were collected at the Photon-In Photon-Out X-ray Spectroscopy (PIPOXS) beamline at the Cornell High Energy Synchrotron Source (CHESS) with ring conditions of 100 mA at 6 GeV. The incident energy was selected using a LN_2_-cooled Si(111) monochromator while harmonic rejection and beam focusing were provided by a pair of Rh-coated focusing mirrors in the hutch. Calibration of the incident energy was achieved using the first inflection point of a Co metal foil (7709.0 eV). The beam intensity before and after the sample was recorded using N_2_-filled ion chambers while the fluorescence intensity from the sample was measured using a four-element Vortex detector. The electrochemical sample cell was placed at 45° relative to the incident beam (∼750 µm × 750 µm at the sample). Scans were collected over the energy range from 7.55 to 8.40 keV and multiple scans were averaged together to improve the signal-to-noise ratio (ten scans).

XAS and EXAFS data were processed using the *Demeter* suite of programs (Ravel & Newville, 2005 ▸). EXAFS data were Fourier transformed over the range of *k* = 2–12 Å^−1^.

### 2.2. Electrochemical cell design software

The electrochemical cell for *in situ* XAS was designed using the free version of the *DesignSpark Mechanical* 5.0 software (https://www.rs-online.com/designspark/mechanical-software). Rsdoc (*DesignSpark Mechanical*) files are provided in the supporting information, which can be used to export into STL files for free using the *DesignSpark Mechanical* software.

### 2.3. Electrochemical cell fabrication

A Genmitsu 3018-PROVer hobbyist CNC machine was used for fabrication of all the parts. *DeskProto* software (https://www.deskproto.com/) was used to obtain the G-code file that was sent to the CNC machine for fabrication. The CNC machine used is based on open-source *GRBL* software (https://github.com/grbl/grbl), which may be used with various free software packages that can send the G-code to the machine, such as the *Universal Gcode Sender* (https://winder.github.io/ugs_website/). Designing the electrochemical cell with free design software and fabrication using a low-cost hobbyist CNC machine ensures high accessibility for researchers to download, modify and fabricate their own electrochemical cell.

### 2.4. Electrochemical cell materials

The electrochemical cell was fabricated using different materials to ensure its versatility in different environments. The *in situ* XAS experiments were made using 0.1 *M* KOH solutions in poly(methyl methacrylate) (PMMA, Plexiglass) and polyether ether ketone (PEEK) electrochemical cells, but other materials can be used for different solutions. Polytetra­fluoro­ethyl­ene (PTFE, Teflon) and polycarbonate (Lexan) cells were also fabricated using the design shown here with the same results. Carbon paper (AvCarb MGL 190) used in the experiments was acquired from Fuel Cell Store with a thickness of 190 µm.

## 3. Results and discussion

**Figure 1:**
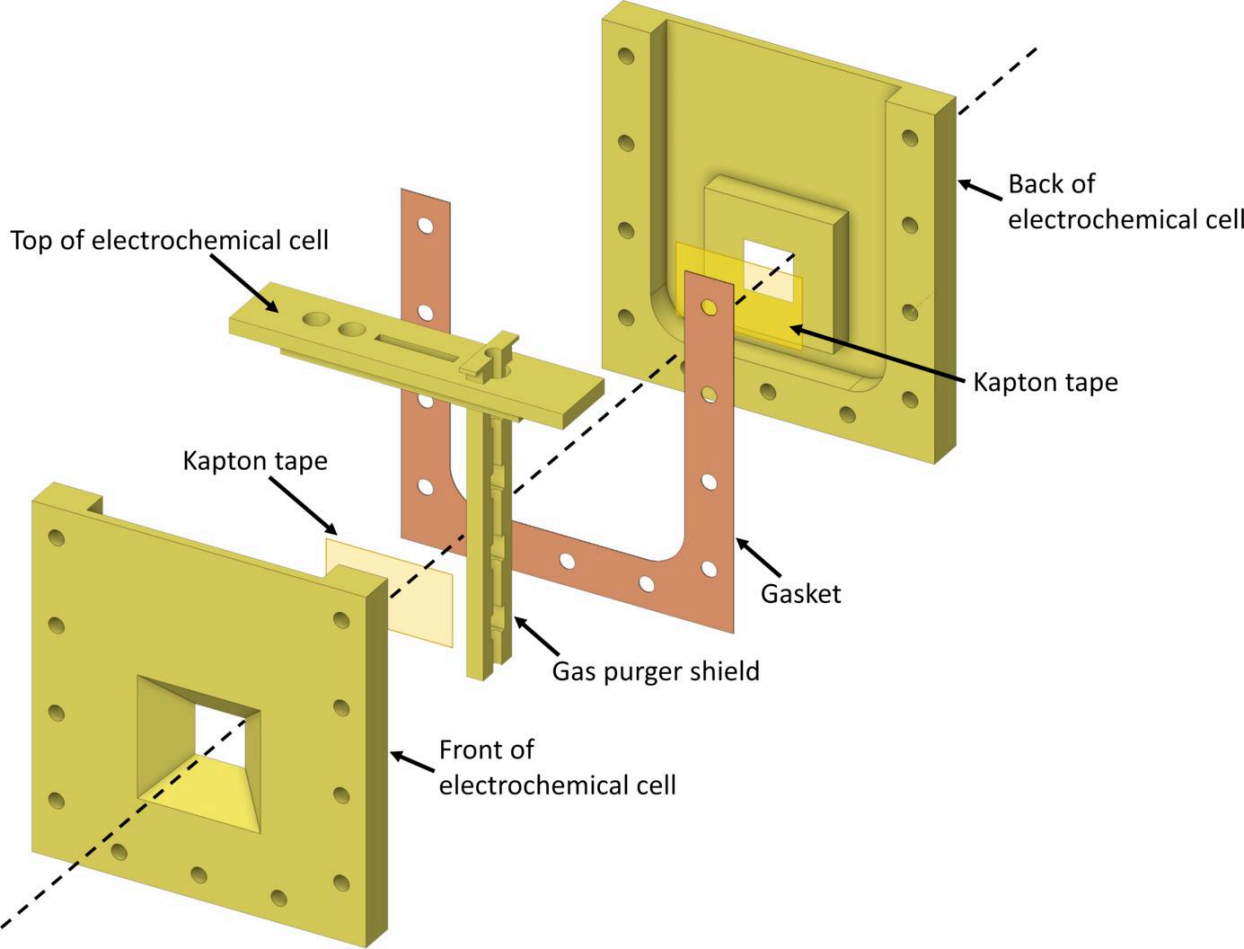
An exploded view of the electrochemical cell.

Leveraging the power of XAS requires presenting an electrochemical system in such a way that an X-ray beam can reach the material of interest and then subsequently also make its way to a detector, either via direct transmission or through fluorescence. While sample cells exist for this purpose, they are often somewhat inflexible, and there are currently no commercially available electrochemical cells amenable to performing XAS in both absorption and fluorescence modes; even recently reported bespoke sample environments lack this capability (Yang *et al.*, 2019 ▸). To address this gap, we have designed a flexible electrochemical cell with the ability to (1) control the thickness of the electrolyte around the working electrode, (2) deliver gas to the electrolyte without interfering with the working electrode or X-ray beam, and (3) collect simultaneous absorption and fluorescence XAS data (Fig. 1 ▸).

This cell houses all three necessary electrodes (working, reference and counter electrodes) within a single unit and allows for gas purging without disrupting any of the electrodes’ functions. In this design, the counter electrode is positioned farthest from the working electrode, while the tip of the reference electrode is closest to the XAS active area. To enable gas purging without interfering with the working electrode or the X-ray beam, plastic tubing has been integrated next to the purging attachment, featuring square holes that promote gas diffusion while covering the gas outlets, ensuring that bubbling does not affect the working electrode’s operation.

Fig. 1 ▸ presents an exploded view of our electrochemical cell. Both external plates incorporate a 45° chamfer into the large (12.7 mm × 12.7 mm) analysis window, ensuring its applicability in both absorption and fluorescence modes. This large opening window enables the use of many spots on the sample, even with unfocused X-ray beams of the order of millimetres^2^. For example, with a beam of 1 mm × 1 mm, this cell would enable access to 84 unique spots on the sample and thus allow data collection even in cases where samples damage rapidly in the incident beam. Kapton tape was employed within each window to prevent solution leakage and allow X-ray access to the sample. The dashed line delineates the axis perpendicular to the cell. The top of the electrochemical cell was similarly manufactured using the same CNC machine and the 3D model is accessible in the supporting information.

To regulate the separation between the front and back plates for electrode/electrolyte thickness adjustment, we employed a manually cut silicon gasket measuring 254 µm in thickness and possessing a 10 A durometer rating (McMaster–Carr part number 86435K41). If a greater separation is necessary between the plates for a thicker working electrode, a thicker gasket or multiple gaskets together can be utilized. These silicon gaskets can either be manually cut or cut using an economical hobbyist laser or blade cutter machine.

At the top of the electrochemical cell, circular openings are designated for the counter (outer hole) and reference electrodes. Adjacent to the reference electrode, a rectangular aperture is allocated for the carbon paper utilized as the working electrode. Introducing the gas-purger shield next to the working electrode, with its circular side oriented against the electrodes, is essential. The plastic tubing is inserted into the circular section of the shield and small incisions are made in the tube at heights corresponding to the covered portions of the shield.

The electrochemical cell was assembled using 11 socket head screws with an 8-32 thread size, each measuring 3/4 inches in length. Additionally, the back plate of the cell underwent threading using an 8-32 tap. We conducted tests on the cell using two types of screws: 18-8 stainless steel (McMaster–Carr part number 92196A197) and glass-filled nylon (McMaster–Carr part number 91221A455). Both types performed effectively. Importantly, because the gasket prevents any leakage, stainless steel or aluminium screws can be employed without the risk of contaminating the solution with metals.

**Figure 2:**
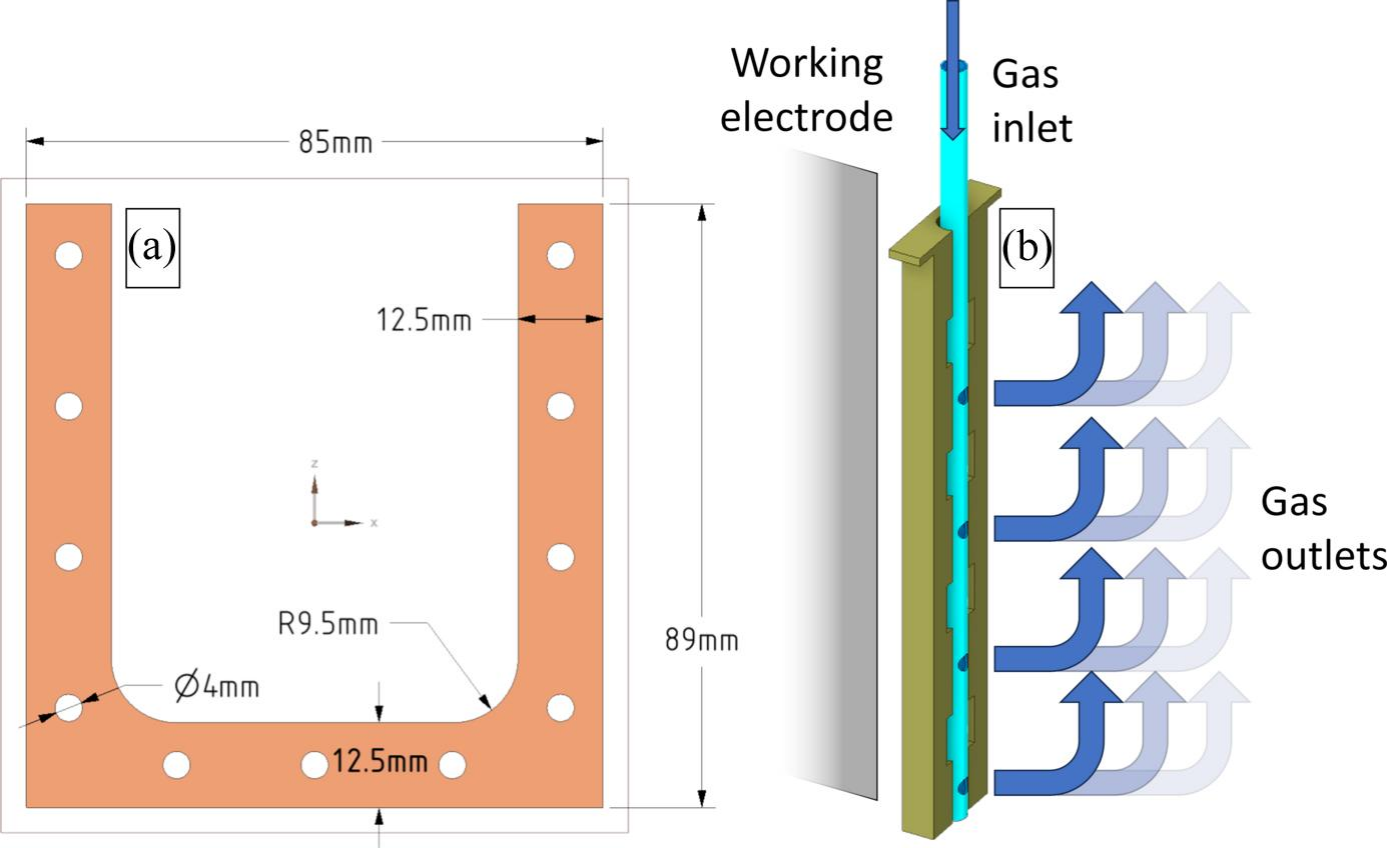
(*a*) Gasket size for the electrochemical cell used and (*b*) a diagram of gas purging to the side contrary to the working electrode to avoid bubbles that would affect XAS measurements.

Fig. 2 ▸(*a*) illustrates the dimensions of the silicon gasket, aligning with the dimensions of the electrochemical cell. We initially tested a cut O-ring to seal the cell; however, this approach led to cell deformation due to uneven pressure distribution, resulting in leaks. Consequently, we recommend using a complete gasket that covers the entire electrochemical cell wall for optimal performance.

In Fig. 2 ▸(*b*), we provide a more detailed view of the purger attachment, highlighting the gas-outlet positions in relation to the shield’s covered sections. The slots in the attachment play a crucial role in ensuring proper gas diffusion within the solution. Additionally, it is vital to position the purger correctly to prevent bubbles from reaching the working electrode, which could lead to measurement artefacts in both electrochemical and XAS data.

**Figure 3:**
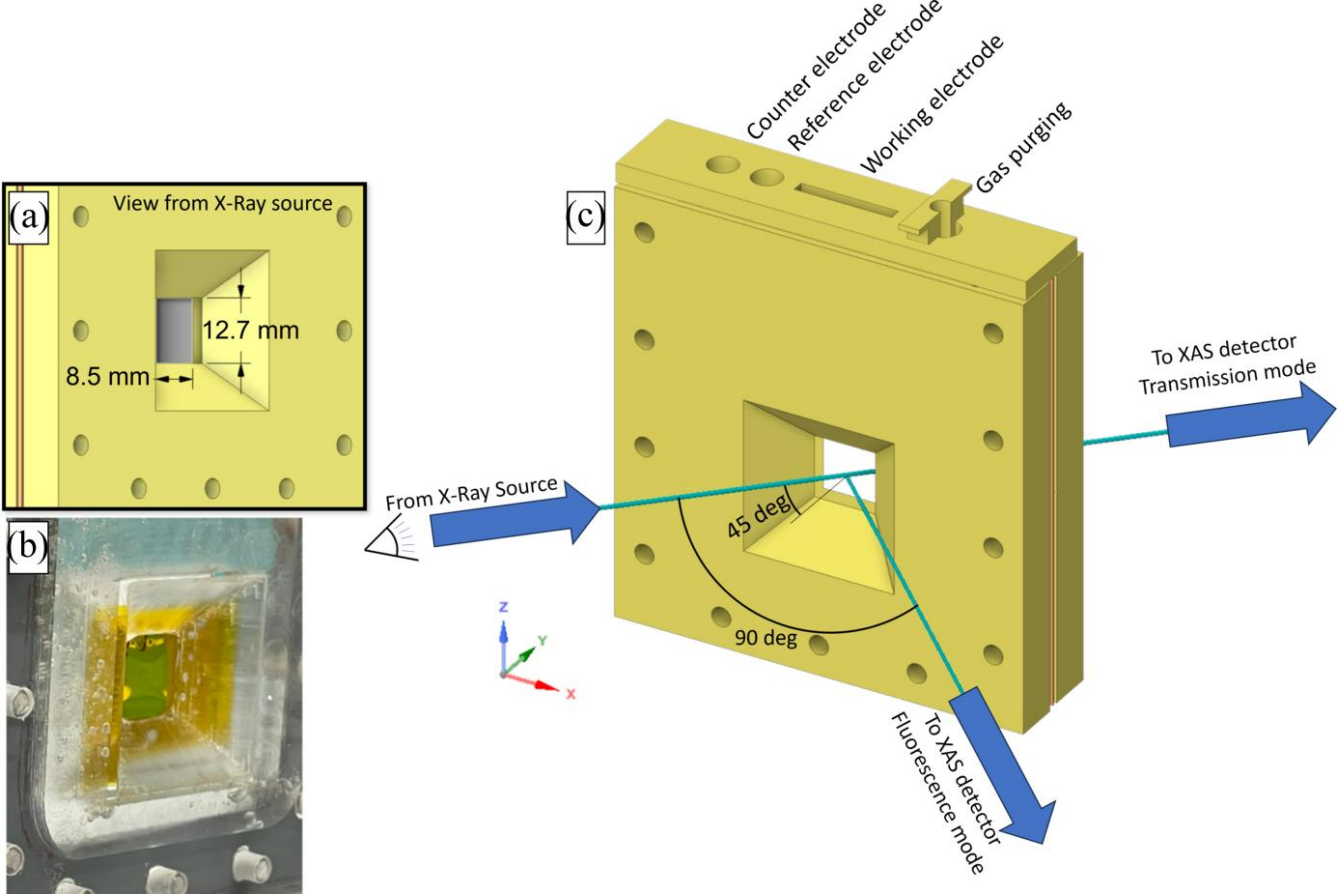
(*a*) *X–Y* in-plane 45° view to the *Y* axis (view from the X-ray source) of the sample using a 254 µm gasket with a sample window of 8.5 mm by 12.7 mm for XAS experiments. (*b*) The same view in the PMMA electrochemical cell used for *in situ* XAS experiments. (*c*) A diagram of the electrochemical cell with positions of electrodes, gas purging, and relative positions for the X-ray source and XAS detectors in transmission and fluorescence modes.

Because this electrochemical cell can be used for XAS measurements in transmission and fluorescence modes, depending on the needs of the user, Fig. 3 ▸(*a*) presents a perspective of the electrochemical cell as viewed from the X-ray source, set at a 45° angle from the normal orientation of the cell window. The electrochemical cell model shown for XAS measurements in transmission mode has a separation between the front and back plates of 254 µm to account for the gasket used in our experiments. The size of the available area for XAS measurements is then 8.5 mm by 12.7 mm, large enough to acquire data at many discrete locations on the electrode even with an unfocused X-ray beam. The horizontally accessible area can change slightly depending on the thickness of the gasket, though in all conceivable cases will permit many millimetres of the sample to be probed. In a worst-case scenario, with a gasket of 2 mm, the available area for XAS measurements would be 7.2 mm by 12.7 mm, still leaving a large area for spectroscopic measurements. Fig. 3 ▸(*b*) shows a picture taken from the X-ray source point of view of the real electrochemical cell fabricated in acrylic at the same 45° angle as in Fig. 3 ▸(*a*). The real cell has corners that depend on the CNC part used for the fabrication, displaying a radius that is not perfectly straight like the model shown. Using carbon paper as the working electrode, AvCarb MGL 190 with a thickness of 190 µm in our case, and the front and back layers of Kapton tape with a thickness of 60 µm each, totals a thickness of 310 µm. We tested various gaskets and determined that employing a 254 µm gasket with separation, attributed to the clearance resulting from the window’s position, ensured a sufficient solution-layer thickness between the tape and the carbon paper. This arrangement allowed for the observation of robust electrochemical behavior without significant X-ray absorption, preserving its suitability for XAS measurements. The full size of the window was selected to be 12.7 mm by 12.7 mm (8.5 mm by 12.7 mm at 45°) because it has been tested previously to work well for electrochemical measurements using carbon paper as the working electrode (Yang *et al.*, 2019 ▸).

In Fig. 3 ▸(*c*), we provide an overview of the entire electrochemical cell, indicating the positions of the X-ray source, fluorescence detector and transmission detector. Depending on the sample concentration, users can opt for either fluorescence and/or transmission detectors without needing to relocate the cell within the electrochemical cell holder. The electrode positions are clearly labeled atop the electrochemical cell for reference.

**Figure 4:**
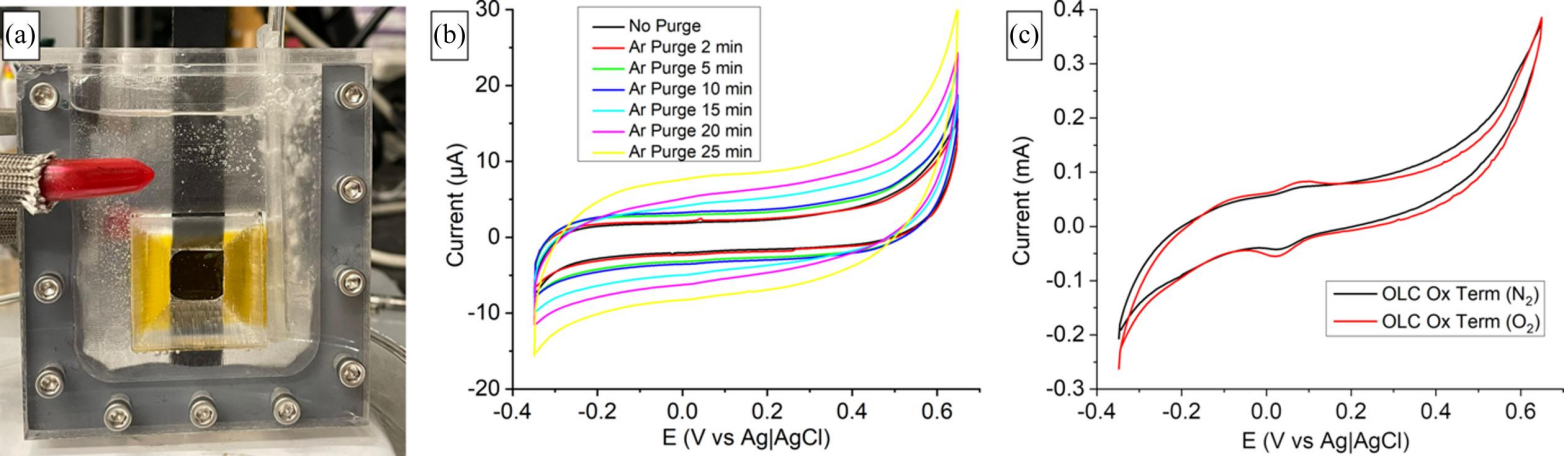
(*a*) The PMMA electrochemical cell with carbon-paper working electrode while purging with argon on the right. (*b*) Cyclic voltammetry of the carbon paper before and after argon purging at different times. (*c*) Cyclic voltammetry of carbon paper after nitrogen and oxygen purging.

Fig. 4 ▸(*a*) shows a picture of the electrochemical cell made of acrylic with carbon paper used as the working electrode and the tube on the right bubbling with argon. These bubbles exit the tube in a direction opposite to the working electrode’s location. This deliberate design choice serves the purpose of preventing any potential issues arising from bubble interference with the measurements. In Fig. 4 ▸(*b*), we present the results of cyclic voltammetry performed on the carbon paper. The experimental setup involved a carbon counter electrode and an Ag|AgCl reference electrode, both immersed in a 0.1 *M* KOH solution. The cyclic voltammetry data exhibited the anticipated behavior characteristic of carbon paper. Notably, there was no discernible increase in ohmic drop, a phenomenon that could arise in the context of a sandwiched cell configuration. To further elucidate the electrochemical response of the carbon paper within the electrochemical cell, we conducted measurements before and after exposing the system to periods of argon purging. These intervals included durations of 2, 5, 10, 15, 20 and 25 min of argon purging. Importantly, no instances of leakage were detected during these experiments. The only change was an increase in double-layer capacitance.

We tested the effect of our purging attachment by measuring its effect on the oxygen reduction reaction (ORR). We introduced an onion-like carbon (OLC) ink to the carbon paper precisely at the center of the window. The cell was purged with dry nitro­gen for 15 min and we obtained the cyclic voltammetry shown in Fig. 4 ▸(*c*) (black). Subsequently, a 15 min purge with oxygen was conducted, and the resulting cyclic voltammetry is presented in Fig. 4 ▸(*c*) (red). The oxidized OLC revealed redox peaks around 0.1 V versus Ag|AgCl, exhibiting a noticeable increase after oxygen purging. Furthermore, an elevation in ORR activity was observed after oxygen purging, evident in the increased cathodic current at negative potentials.

With the electrochemical fidelity of the cell established, we next tested it for *in situ* XAS measurements. The electrochemical cell was placed at a 45° angle with respect to the X-ray source and XAS measurements were made in fluorescence mode (alignment was performed using transmission and fluorescence simultaneously). OLC nanoparticles were synthesized, and iron and cobalt (1:3) were deposited on their surface at 20%(*w*/*w*) with respect to carbon. This material was deposited on the carbon paper by drop-coating in the area exposed for XAS measurements in the electrochemical cell window. A solution containing 0.1 *M* KOH with a pH of 13.05 was employed. Two chronoamperometry experiments were performed, both at 0.4 V versus reversible hydrogen electrode (RHE), with and without the purging of oxygen gas for a duration of 15 min to saturate the solution. Having the reference electrode next to the working electrode, we were able to decrease the uncompensated resistance of the cell. While the potential was applied, XAS data were obtained in fluorescence mode.

**Figure 5:**
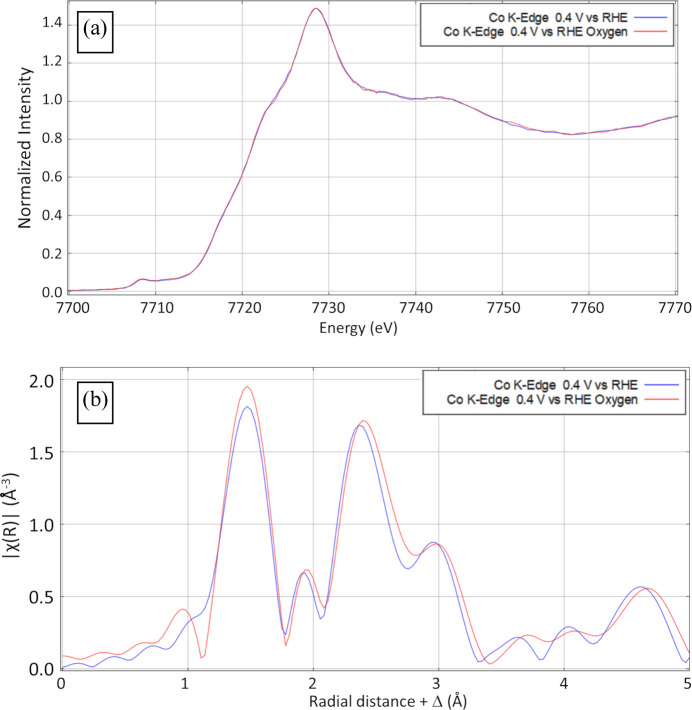
XAS measurement of the Co *K*-edge of OLC/FeCo at different potentials, before and after bubbling with oxygen, showing (*a*) normalized intensity and (*b*) Fourier transformed EXAFS (*k* = 2–12 Å^−1^) spectra in *R* space.

Fig. 5 ▸(*a*) displays the XANES spectra from both experiments while the Fourier transformed EXAFS data are displayed in Fig. 5 ▸(*b*) (*k*-space EXAFS data are shown in Fig. S1 of the supporting information). The data are consistent with Co in the +2 oxidation state (Aðalsteinsson *et al.*, 2020 ▸; Chou *et al.*, 2020 ▸) and suggest Co–O and Co–Co scatterers at the distances expected for CoO (Chou *et al.*, 2020 ▸). Importantly, within experimental uncertainty, the results for the samples with and without O_2_ purging are identical and demonstrate that gas purging does not negatively impact the experiment. The displayed data were collected in an average of 11.3 min per scan, with all ten scans for each experiment taking ∼112 min, confirming that quality EXAFS data may be obtained in reasonable experimentally relevant acquisition times.

## 4. Conclusions

We designed and constructed an open-source electrochemical cell for conducting *in situ* XAS and electrochemistry experiments simultaneously. This cell houses all three electrodes within the same chamber, with the reference electrode positioned closest to the working electrode. We also created an attachment that facilitates the purging of gas into the solution. Special shields within the attachment guide the bubbles away from the working electrode, preventing them from interfering with XAS and electrochemical measurements. The electrochemical cell features windows on both sides covered with Kapton tape, each with 45° chamfers, providing 7.2 mm by 12.7 mm of available space for XAS in transmission and/or fluorescence mode depending on the sample concentration, without changing the cell or entering the experimental hutch. We conducted tests on the electrochemical cell, both with and without gas purging, and observed no indication of an elevated ohmic drop. Concurrent *in situ* XAS/electrochemistry experiments were performed showing no differences between samples, demonstrating that gas purging does not cause any artefacts in the XAS data. A crucial aspect of our electrochemical cell design is its accessibility and affordability, making it feasible for anyone to fabricate using easily obtainable low-cost components. The schematic design was created using the free version of the *DesignSpark Mechanical* software, and the original files (rsdoc files) for each part are included in the supporting information. All parts were manufactured using an economical CNC 3018 machine controlled by *GRBL*-based software.

## Supplementary Material

Supporting information. DOI: 10.1107/S1600577524000122/vy5020sup1.pdf

Electrochemical cell main. DOI: 10.1107/S1600577524000122/vy5020sup2.zip

Electrochemical cell purger. DOI: 10.1107/S1600577524000122/vy5020sup3.zip

Electrochemical cell top. DOI: 10.1107/S1600577524000122/vy5020sup4.zip

Electrochemical cell gasket. DOI: 10.1107/S1600577524000122/vy5020sup5.zip
