# Supplementary material for: Open-source electrochemical cell for in situ X-ray absorption spectroscopy in transmission and fluorescence modes
Source: J Synchrotron Radiat. 2024 Feb 2;31(Pt 2):322–7. doi: 10.1107/S1600577524000122 (PMC10914171; doi:10.1107/S1600577524000122)
Supplement: Supplementary file 1 [file s-31-00322-sup1.pdf]

JOURNAL OF SYNCHROTRON RADIATION

Volume 31 (2024)

Supporting information for article:

Open-source electrochemical cell for *in situ* X-ray absorption spectroscopy in transmission and fluorescence modes

Hiram Lopez-Astacio, Brenda Lee Vargas-Perez, Angelica Del Valle-Perez, Christopher J. Pollock and Lisandro Cunci

S1. Fourier Transformed EXAFS Spectrum

Figure S1 shows the Fourier transformed EXAFS data over the range of k = 2 – 12 $Å^{-1}$ for both experiments, before and after purging with oxygen for 15 minutes. Data is very similar, showing no artifacts due to gas purging using the electrochemical cell.

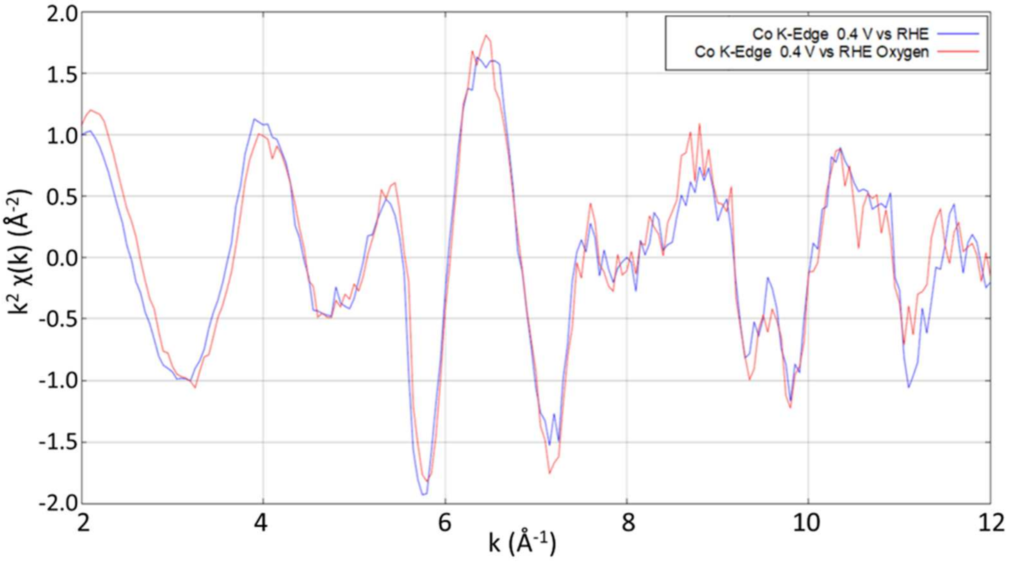

Figure S1 Fourier transformed EXAFS data before and after purging with oxygen for 15 minutes.

S2. Files in Supplementary Information

S2.1. Electrochemical Cell Main.rsdocx

- This file contains the main sides of the electrochemical cell with a gasket. The user only needs to print one of the two sides twice. The file shows a 3D design of the assembled electrochemical cell.

S2.2. Electrochemical Cell Purger.rsdocx

- This file contains the gas purger that is placed from the top side of the electrochemical cell.

S2.3. Electrochemical Cell Top.rsdocx

- This file contains the 3D design of the electrochemical cell top. The size of the holes fit common reference and counter electrodes (6 mm diameter) but can be modified.

S2.4. Electrochemical Cell Gasket.rsdocx

- This file contains the design of the gasket with the dimensions annotated.
