# Supplementary figures and images for: Open-source electrochemical cell for in situ X-ray absorption spectroscopy in transmission and fluorescence modes
Source: J Synchrotron Radiat. 2024 Feb 2;31(Pt 2):322–7. doi: 10.1107/S1600577524000122 (PMC10914171; doi:10.1107/S1600577524000122)

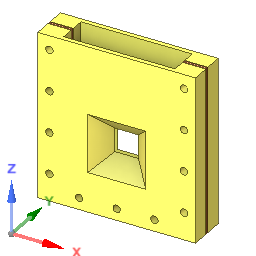

Supplement: Supplementary file 2 [file s-31-00322-sup2.zip › docProps/thumbnail.png]

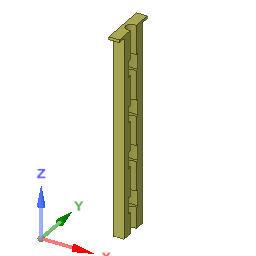

Supplement: Supplementary file 3 [file s-31-00322-sup3.zip › docProps/thumbnail.png]

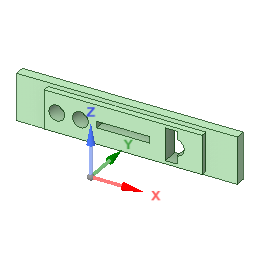

Supplement: Supplementary file 4 [file s-31-00322-sup4.zip › docProps/thumbnail.png]

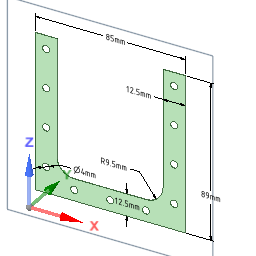

Supplement: Supplementary file 5 [file s-31-00322-sup5.zip › docProps/thumbnail.png]
